# Preoperative glasgow prognostic score was an effective prognostic indicator in patients with biliary tract cancer

**DOI:** 10.3389/fimmu.2025.1560944

**Published:** 2025-04-08

**Authors:** Rongqiang Liu, Ling Wang, Jing Ye, Xinyi Li, Wangbin Ma, Ximing Xu, Jia Yu, Weixing Wang

**Affiliations:** ^1^ Department of Hepatobiliary Surgery, Renmin Hospital of Wuhan University, Wuhan, Hubei, China; ^2^ Cancer Center, Renmin Hospital of Wuhan University, Wuhan, Hubei, China

**Keywords:** glasgow prognostic score, prognosis, biliary tract cancer, meta-analysis, survival

## Abstract

**Background:**

The Glasgow Prognostic Score (GPS) is a well-established prognostic indicator that effectively reflects the inflammatory, nutritional, and immune status of cancer patients. GPS has been shown to be associated with survival outcomes in many different cancers. However, its prognostic significance in biliary tract cancer (BTC) remains unclear. This meta-analysis aims to explore the prognostic value of GPS in BTC patients.

**Methods:**

A systematic search was conducted in PubMed, Embase, and Web of Science to identify relevant studies. Survival data including overall survival (OS), disease-free survival (DFS) and recurrence-free survival (RFS) were the main observation indicators. Hazard ratios (HRs) with 95% confidence intervals (CIs) were extracted and pooled for meta-analysis.

**Results:**

A total of 16 articles incorporating 1919 patients were included in the study. High GPS was associated with poor OS (HR:2.00, 95% CI:1.62-2.48) and DFS/RFS (HR:2.50, 95% CI:1.71-3.65). Subgroup analysis further confirmed the prognosis value of GPS in BTC patients.

**Conclusions:**

GPS could serves as a valuable prognostic marker in BTC patients and may aid in risk stratification and treatment decision-making.

## Introduction

Cancers has exceeded other diseases as the leading threat to human health (1). Biliary tract cancer (BTC) is a highly malignant tumor characterized by an insidious onset and late-stage diagnosis. BTC comprises intrahepatic cholangiocarcinoma (iCCA), extrahepatic cholangiocarcinoma (eCCA), gallbladder cancer (GBC), and periampullary cancer (2). The pathogenesis and behavior of BTC vary across different regions, mainly affected by parasitic infection, chronic inflammation, cholelithiasis, viral infection, genetic factors and environmental factors (2, 3). In Southeast and East Asia, high prevalence of BTC is closely associated with bile duct parasites and chronic gallstone disease, whereas in Europe and North America, primary sclerosing cholangitis, inflammatory bowel disease and obesity are the primary risk factors (4). South America has a high incidence of GBC, while in Africa, schistosomiasis contributes significantly to BTC incidence (4). Notably, the global incidence of BTC is rising (5). Radical surgery remains the most important treatment method for BTC patients (6). Other therapies such as radiotherapy, chemotherapy and immunotherapy play critical roles in prolonging survival for BTC patients (7–12).

Cancer patients are often accompanied with malnutrition and systemic inflammation (13). The Glasgow Prognostic Score (GPS) has emerged as a novel prognostic tool, effectively reflecting the inflammatory and nutritional status of the cancer patients (14, 15). Several previous meta-analyses have demonstrated the significant prognostic significance of the GPS in urological and gynecologic cancers (16, 17). GPS and its modified counterparts, including the modified GPS (mGPS) were widely used inflammatory indices in clinical practice. A previous meta-analysis demonstrated the prognostic value of mGPS in BTC (18). Nevertheless, the prognostic value of GPS in BTC remained unclear. Several epidemiological studies found that GPS was associated with poor prognosis in BTC patients (19, 20). However, other studies suggested that GPS had no clear relationship with BTC (21, 22). To address this inconsistency, we conducted a comprehensive meta-analysis to evaluate the prognostic significance of GPS in BTC patients.

## Methods

### Search strategy

Three investigators conducted independently systematically searched the PubMed, Embase, and Web of Science databases for the related articles investigating the prognostic value of GPS in BTC. The search deadline was August 20, 2023. The search keywords were utilized: (bile duct adenoma OR bile duct neoplasms OR bile duct cancer OR bile duct tumor OR cholangiocarcinoma OR cholangiocellular carcinoma OR gallbladder cancer OR gallbladder carcinoma) AND (glasgow prognostic score OR GPS). There were no language restrictions. References of included studies were manually screened for additional relevant articles. We implemented this meta-analysis according to the PRISMA guidelines.

### Study selection

Inclusion criteria were as follows:(1) evaluated the correlation between GPS and the prognostic significance of BTC. (2) adequate data was utilized to analyze the hazard ratios (HRs) and 95% 95% confidence intervals (CIs). Exclusion standards were as follows: studies with inadequate data, duplicated data, letters, reviews or abstracts.

### Data extraction and quality assessment

Three investigators independently extracted the following data: first author, publication year, study design, country, sample size and survival outcomes. Study quality was assessed using the Newcastle-Ottawa Scale (NOS) (23). If the study did not provide survival data directly, we utilized engage Digitizer version 4.1 to obtain survival data from the survival curve according to Tierney method (24).

### Data analysis

HRs and corresponding 95% CIs were used to analyze the prognosis value of GPS in BTC. Heterogeneity was assessed using I^2^. We utilized a fixed-effects model if I^2^ <50%, and a random-effects model was employed if I^2^>50%. Subgroup analysis was performed to further test the prognostic value of GPS. Meta-regression was applied to search the source of heterogeneity. Sensitivity analysis was utilized to assess the stability of the outcomes. Begg’s test, Egger’s test and the trim-and-fill method were applied to evaluate the publication bias. All data analyses was performed using STATA 12.0 (STATA Corp., College Station, TX, USA).P <0.05 was statistically significant.

## Results

### Search results

**Figure 1:**
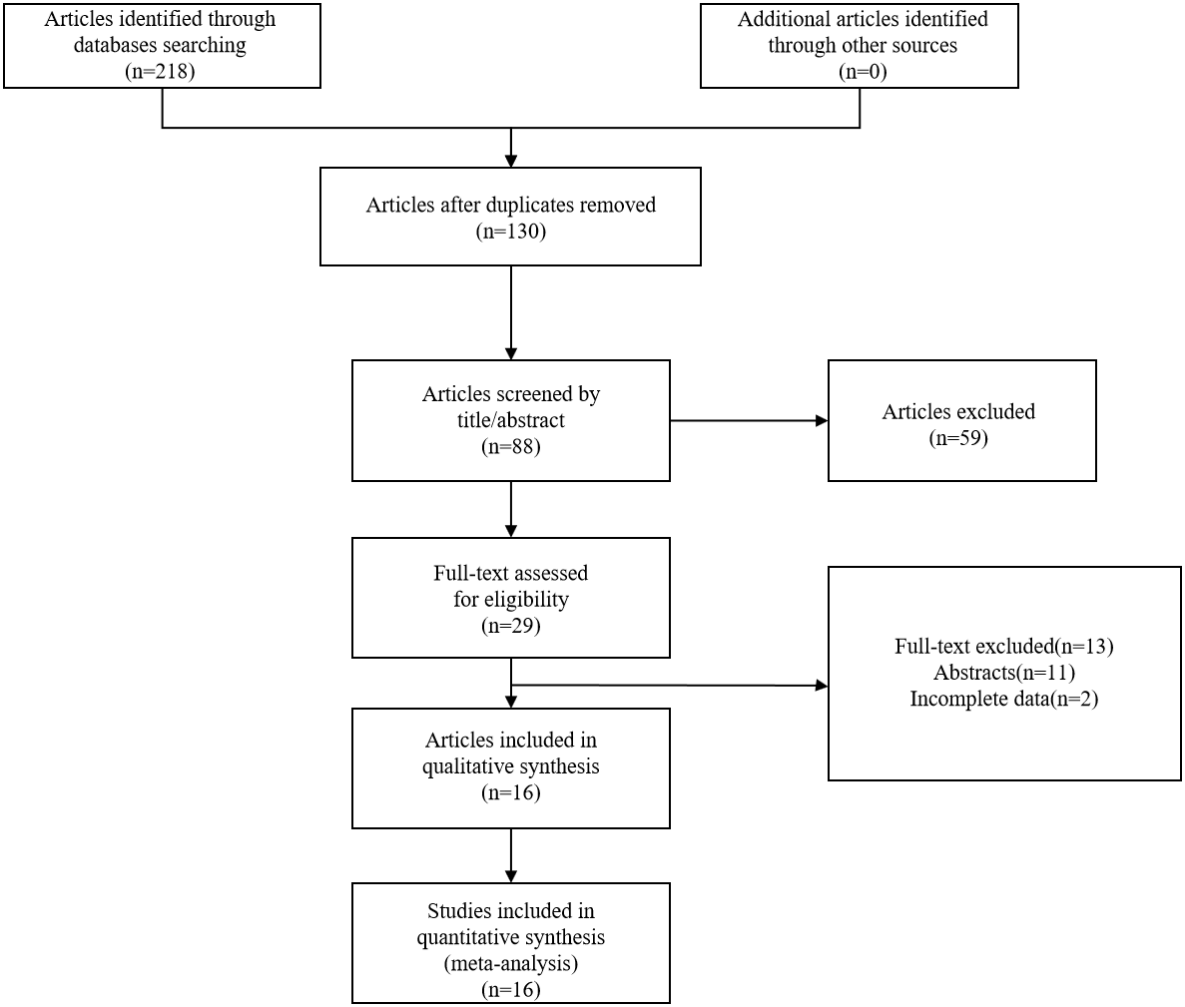
The flow chart for study selection.

Through a systematic search, we initially identified 218 articles. After the deletion of 130 duplicated articles, 88 articles were retained. After screening titles and abstracts, 72 papers that did not meet the inclusion criteria were removed. Finally,16 articles were included in the meta-analysis (19–22, 25–36). The study selection process was illustrated in **Figure 1**.

### Study characteristics

A total of 16 retrospective studies comprising 1919 patients were enrolled in the meta-analysis. 8 studies were performed in Japan. 6 studies were from China. The other studies were from Italy and Sweden. The NOS scores of all incorporated studies were greater than 5. The characteristics of the included studies were displayed in **Table 1**.

**Table 1:** Basic characteristic of the included studies.

Study	Year	Country	Study type	Sample	Treatment methods	Analysis type	Survival analysis	NOS score	Histological type
Asakura	2022	Japan	R	169	Surgery	UVA	OS	6	eCCA
Conci	2021	Italy	R	282	Surgery	UVA	OS	6	BTC
Fujiwara	2019	Japan	R	121	Surgery	MVA	OS,DFS	7	eCCA
Hoshimoto	2019	Japan	R	43	Surgery	MVA	OS,DFS	7	eCCA
Hu	2018	China	R	173	Surgery	UVA	OS	6	eCCA
Iwaku	2014	Japan	R	52	chemotherapy	MVA	OS	6	BTC
Jansson	2020	Sweden	R	168	Surgery	MVA	OS	6	BTC
Matsumoto	2020	Japan	R	72	Surgery	UVA	OS, RFS	7	iCCA
Moriwaki	2014	Japan	R	62	chemotherapy	MVA	OS	6	BTC
Oshiro	2013	Japan	R	62	Surgery	MVA	OS	6	eCCA
Pan	2017	China	R	72	Surgery	MVA	OS,DFS	7	iCCA
Shiba	2013	Japan	R	30	Surgery	MVA	OS,DFS	7	eCCA
Sui	2020	China	R	273	Surgery	MVA	OS	6	iCCA
Yang	2022	China	R	73	anti-PD-1 therapy	UVA	OS	6	iCCA
Lin	2019	China	R	123	Surgery	UVA	OS	6	iCCA
Bao	2020	China	R	144	Surgery	MVA	OS	6	GBC

R, retrospective; OS, overall survival; DFS, disease-free survival; RFS, recurrence free survival; MVA, multivariate analysis; UVA, univariate analysis; iCCA, intrahepatic cholangiocarcinoma; eCCA, extrahepatic cholangiocarcinoma; GBC, gallbladder cancer; BTC, bile duct cancer.

### Association between high GPS and OS

**Figure 2:**
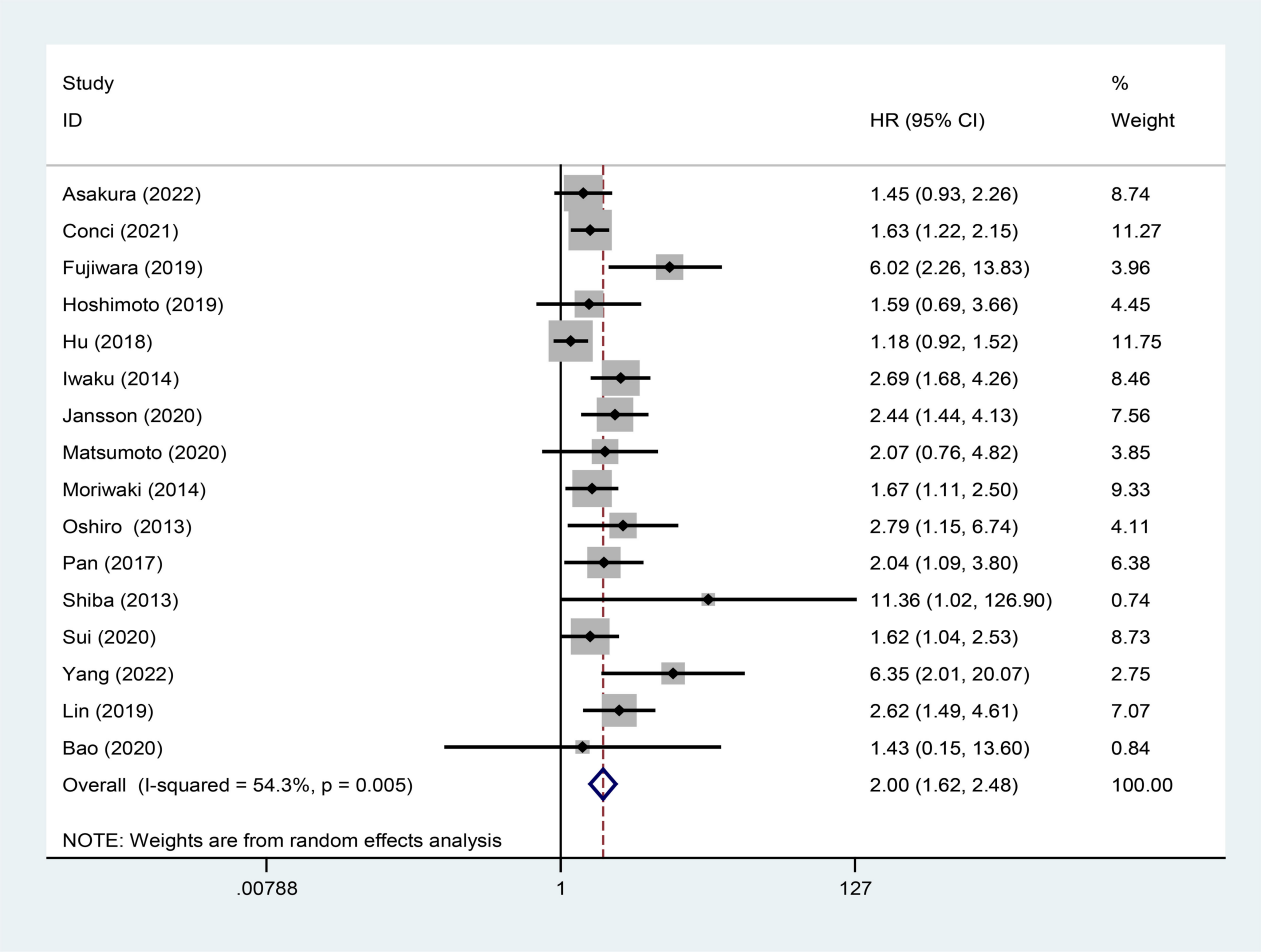
Forest plot of the relationship between high GPS and OS. GPS, glasgow prognostic score; OS, overall survival.

16 studies assessed the association between high GPS and OS. Due to moderate heterogeneity (I^2^ = 54.3%), a random effects model was utilized. The pooled analysis revealed that high GPS was significantly correlated with worse OS (HR:2.00, 95% CI:1.62-2.48) (**Figure 2**).

### Subgroup analysis and meta-regression for OS

Subgroup analysis and meta-regression were performed based on country, treatment method, histological type and analysis type (**Table 2**). We discovered that high GPS had better prognostic value for iCCA and eCCA. As for the other subgroups, the results indicated that high GPS was a poor prognostic factor. Moreover, meta-regression suggested that histological type could be the source of heterogeneity.

**Table 2:** Subgroup analysis and meta-regression for OS.

Factors	Studies	HR(95%CI)	P	Heterogeneity		Meta-regression		
				I^2^	P	Tau^2^	Adj R^2^ (%)	P
**Country**						0.09	-18.52	0.745
Japan	8	2.04(1.64-2.54)	<0.01	45.4	0.077			
China	6	1.92(1.27-2.89)	0.002	64.8	0.014			
Italy	1	1.628(1.217-2.153)						
Sweden	1	2.44(1.44-4.13)						
Treatment method								
Surgery	13	1.89(1.50-2.38)	<0.01	50	0.02	0.08	-4.02	0.39
Non-surgery	3	2.53(1.43-4.48)	0.001	65.2	0.057			
Histological type								
iCCA	5	2.12(1.60-2.81)	<0.01	26.7	0.244	0.031	80.25	0.047
eCCA	6	2.05(1.24-3.39)	0.005	70.5	0.005			
BTC	3	1.89(1.56-2.29)	<0.01	33.7	0.21			
GBC	1	1.435(0.151-13.602)						
Analysis type								
MVA	10	2.13(1.75-2.59)	<0.01	24.6	0.217	0.055	-27.72	0.29
UVA	6	1.76(1.27-2.42)	0.001	64.2	0.016			

MVA, multivariate analysis; UVA, univariate analysis; iCCA, intrahepatic cholangiocarcinoma; eCCA, extrahepatic cholangiocarcinoma; GBC, gallbladder cancer; BTC, bile duct cancer.

### Association between high GPS and DFS/RFS

**Figure 3:**
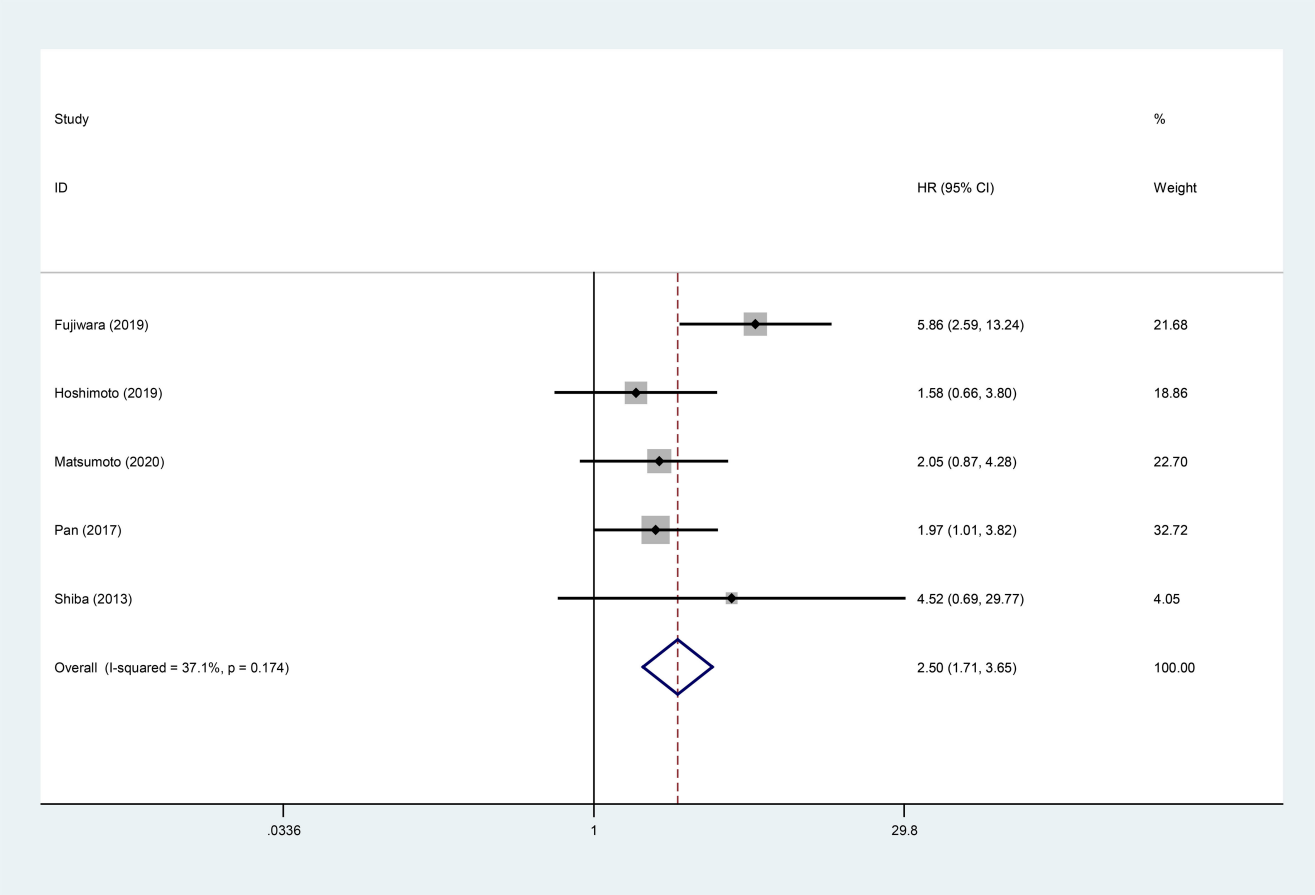
Forest plot of the relationship between high GPS and DFS/RFS. GPS, glasgow prognostic score; DFS/RFS, disease-free survival/recurrence-free survival.

5 studies displayed the relationship between high GPS and DFS/RFS. The meta-analysis showed that high GPS was associated with worse DFS/RFS (HR:2.50, 95% CI:1.71-3.65) (**Figure 3**).

### Sensitivity analysis and publication bias

**Figure 4:**
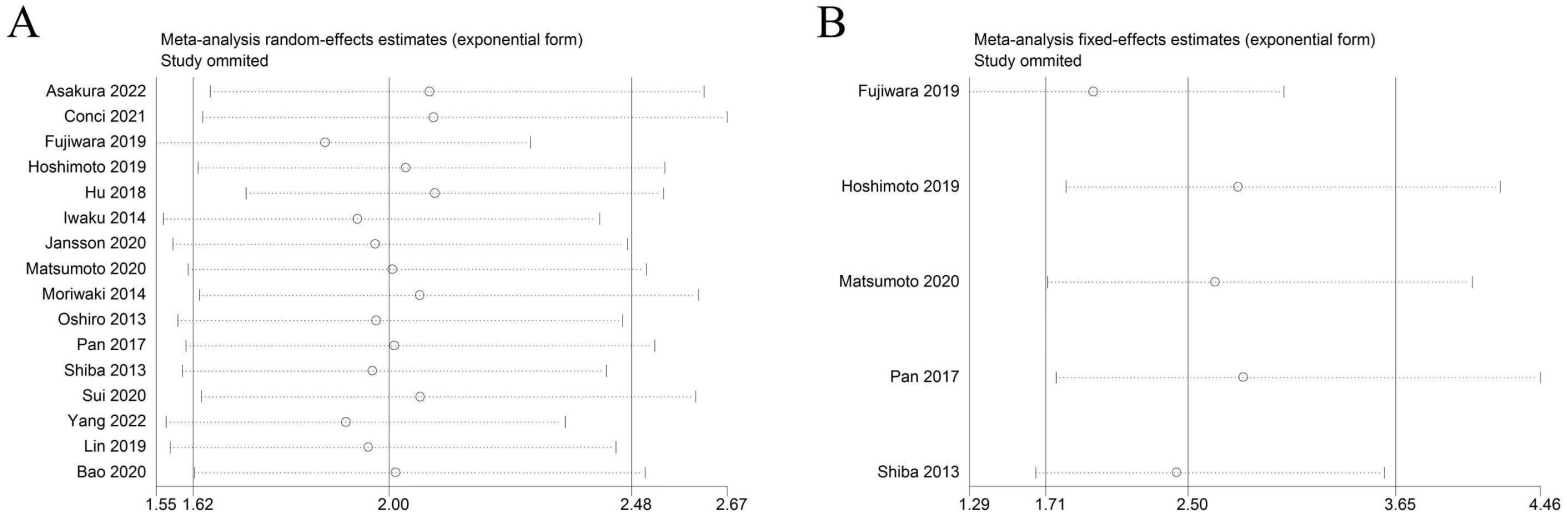
Sensitivity analysis. **(A)** sensitivity analysis for OS. **(B)** sensitivity analysis for DFS/RFS. GPS, glasgow prognostic score; OS, overall survival;DFS/RFS, disease-free survival/recurrence-free survival.

**Figure 5:**
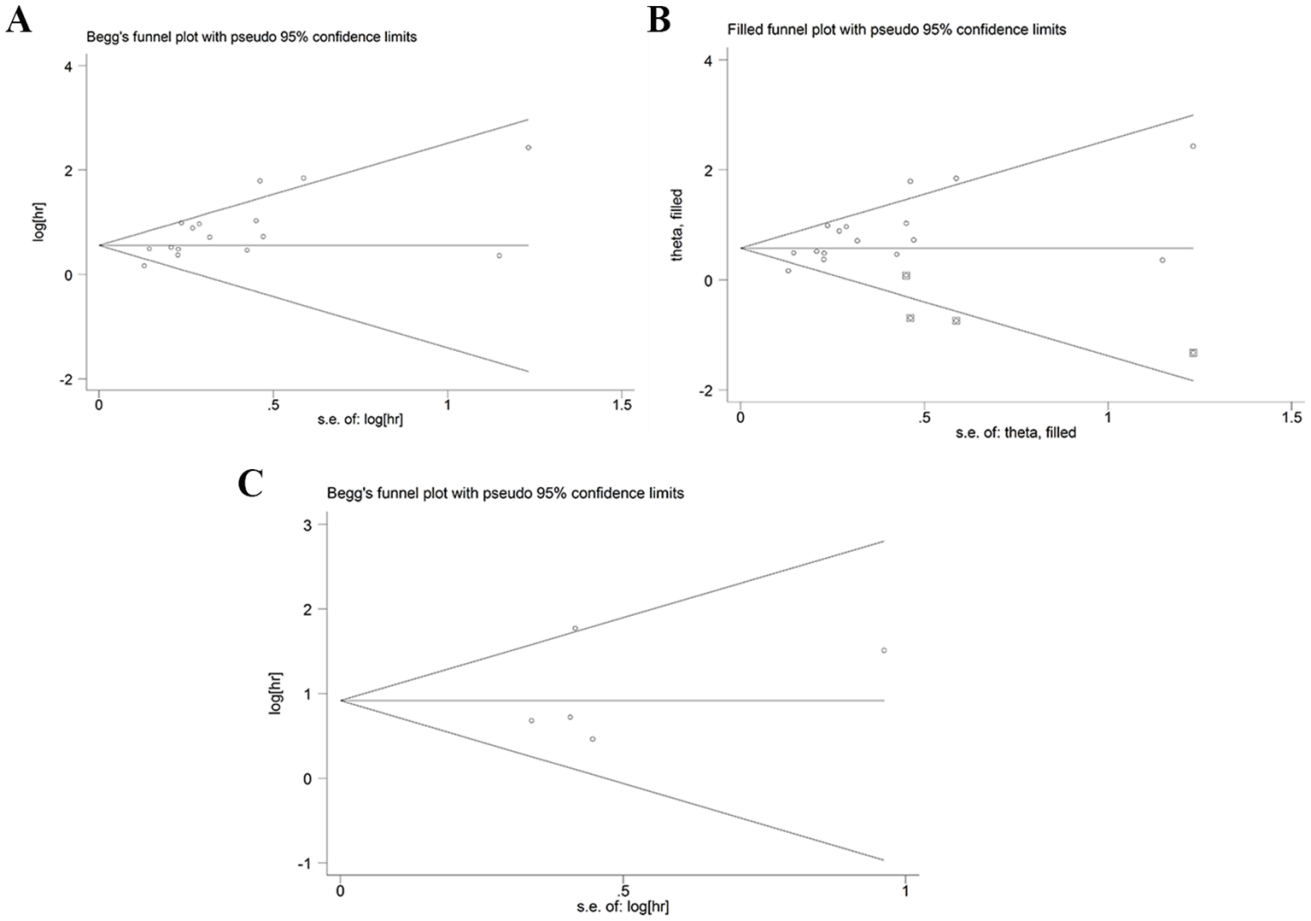
Publication bias. **(A)** publication bias for OS. **(B)** trim-and-fill method for OS. **(C)** publication bias for DFS/RFS. GPS, glasgow prognostic score; OS, overall survival; DFS/RFS, disease-free survival/recurrence-free survival.

Sensitivity analysis was used to detect the stability of the outcomes. The results were consistent with the comprehensive analysis, which confirmed that the results of meta-analysis were stable (**Figures 4A, B**). Begg’s and Egger’s tests were utilized to appraise publication bias. The p values of Begg’s and Egger’s tests for OS were 0.022 and 0.003 (**Figure 5A**), respectively. The trim-and-fill method demonstrated that the outcome for OS was not influenced by the bias (HR: 1.786, 95CI%:1.424-2.239) (**Figure 5B**). The p values of Begg’s and Egger’s tests for DFS/RFS values were 0.806 and 0.634 (**Figure 5C**), respectively. No publication bias was detected for DFS/RFS.

## Discussion

Inflammation-based prognostic scores, such as the C-reactive protein-to-albumin ratio and systemic immune-inflammation index have been successfully used to predict the prognosis of BTC (37, 38). However, their widespread clinical application remains limited due to various constraints. Given the aggressive nature and poor prognosis of BTC, identifying simple and more effective prognostic indicators could significantly enhance the stratified management and treatment of BTC patients (39).

Forrest et al. firstly analyzed the prognosis value of GPS in lung cancer (40). Subsequently, the prognosis value of GPS was demonstrated in different cancers. However, the prognostic value of GPS in BTC has not been clarified. To our knowledge, our study was the first meta-analysis to discuss the prognostic impact of GPS in BTC patients. Our findings demonstrated that high GPS was significantly associated with worse OS and DFS/RFS in BTC. Subgroup analysis further confirmed the prognosis value of GPS in BTC patients. Additionally, sensitivity analysis and the trim-and-fill method proved the robustness and reliability of our results. The determination of the prognostic value of GPS in BCT would strongly support the practical application of GPS and its modified counterparts.

In terms of different regions, most of the studies included in this meta-analysis were conducted in Asia, with only one study focusing on Sweden. Therefore, the applicability of the conclusions to other regions or races remained to be further verified. In the current analysis, the majority of BTC patients had undergone surgical resection. The results showed that patients with high GPS had significantly worse OS. While early-stage BTC patients typically undergo surgery, many BTC patients were diagnosed at an advanced stage, precluding surgical intervention. Only three studies in our study evaluated the predictive value of advanced BTC in receiving palliative care. The potential predictive value of GPS in patients with advanced BTC remained to be further investigated. In addition, for BTC subtypes, we found that high GPS had better predictive value for iCCA and eCCA. However, GPS did not appear to have prognostic significance in GBC. Given that only one study focused on GBC, the finding may lack reliability, and more studies about the prognostic value of GPS in GBC were needed to further evaluate their relationship.

GPS is composed of serum C-reactive protein (CRP) and albumin. CRP is an acute-phase protein regulated by IL-6, IL-8, and tumor necrosis factor α (41). Elevated serum CRP levels indicated increased various inflammatory cytokines. Studies have shown that IL-6 could promote tumor proliferation, invasion and metastasis (42). In addition, CRP can suppress tumor lymphocyte activation and promote tumor immunosuppression (43). Studies have also demonstrated that CRP could directly promoted tumor cell proliferation, invasion and migration (44). Therefore, elevated CRP levels could indicate the significant systemic inflammatory response and impaired immune system. High CRP levels could be associated with adverse prognosis in different tumors (45).

Albumin is an important component of human plasma and can effectively reflect the nutritional and immune status of patients with cancer. Hypoproteinemia can lead to multiple immune cell dysfunction and subsequent immunosuppression (46, 47). Furthermore, hypoproteinemia could also promote the progression of cachexia in cancer patients (48). Studies have confirmed that albumin functions as an antitumor factor, directly inhibiting tumor cell proliferation (49). Moreover, albumin could inhibit inflammation by clearing reactive oxygen species and inhibiting oxidative stress (50). Accumulating evidences have suggested that hypoalbuminemia is a negative prognostic factor in various cancers (51–53).

Taken together, the high GPS, characterized by elevated CRP and low albumin levels, reflects significant systemic inflammation, malnutrition, and immune suppression. These fact may efficiently explain why high GPS was associated with poor prognosis in BTC patients.

Several limitations of our study should be acknowledged. Firstly, all articles were retrospective studies. Secondly, the survival data of 2 studies was obtained from the survival curves. They may not equate the actual value. Thirdly, publication bias was observed for OS. Fourthly, we did not evaluate the association between high GPS and clinicopathological characteristics due to lack of data. Finally, most of the studies were from Asia, which may affect the universality of the outcomes. More studies from different countries and regions were warranted.

Despite these shortcomings, our study also had merits. Firstly, the study was the first meta-analysis to explore the prognosis value of GPS in BTC. Secondly, sensitivity analysis confirmed the stability and reliability of our results. Thirdly, the trim-and-fill method verified that publication bias did not significantly affect our conclusions. Fourthly, subgroup analysis further supported the prognosis value of GPS. Finally, GPS can dynamically monitor the prognosis and treatment effect of BCT.

In conclusion, we found that high GPS predicted poor prognosis in patients with BTC. GPS can serve as a valuable prognostic marker for BTC, aiding in the identification of high-risk patients and facilitating personalized treatment strategies. Given the limitations of our study, well-designed, large-scale randomized controlled trials were needed to further validate our findings and explore the clinical utility of GPS and its modified counterparts in BTC.

## Data Availability

The original contributions presented in the study are included in the article/supplementary material. Further inquiries can be directed to the corresponding authors.
